# Innovative metal oxides (CaO, SrO, MgO) impregnated waste-derived activated carbon for biohydrogen purification

**DOI:** 10.1038/s41598-023-31723-4

**Published:** 2023-03-22

**Authors:** Wanida Koo-amornpattana, Poomiwat Phadungbut, Naphaphan Kunthakudee, Woranart Jonglertjunya, Sakhon Ratchahat, Mali Hunsom

**Affiliations:** 1 Department of Chemical Engineering, Faculty of Engineering, Mahidol University, 25/25 Phuttamonthon 4 Road, Salaya, Phuttamonthon, Nakhon Pathom, 73170 Thailand; 2 Associate Fellow of Royal Society of Thailand (AFRST), Bangkok, 10300 Thailand

**Keywords:** Engineering, Materials science

## Abstract

In this work, a series of innovative metal oxide impregnated waste-derived activated carbons (MO/AC) was synthesized and used to purify the simulated biohydrogen based on the concept of CO_2_ removal from the gas stream. Effects of metal oxide types (CaO, SrO and MgO) and contents of the best metal oxides on the morphology and the CO_2_ adsorption capacity from the biohydrogen were investigated. It was found that both metal oxide types and contents played an important role on the adsorbent textural property and surface chemistry as well as the CO_2_ adsorption capacity. Among all synthesized adsorbent, the MgO-impregnated AC with 12 wt.% MgO (12MgO/AC) exhibited the highest CO_2_ adsorption capacity of around 94.02 mg/g. With this successive adsorbent, the biohydrogen with the H_2_ purity higher than 90 mol% can be achieved from the gas stream with 50 mol% CO_2_ for the first 2 min of adsorption period in a fixed bed reactor. The mechanism of CO_2_ adsorption occurred via a combined process of the physisorption and chemisorption. Besides, the 12MgO/AC exhibited a high recyclability after several repetitive adsorption/desorption cycles.

## Introduction

The production of H_2_ via the biological process is one of the currently hottest issues in the field of bioenergy because of its low energy requirement in comparison with the production of H_2_ from fossil fuels. Typically, there are three classical biological processes used to produce the biohydrogen including bio-photolysis, photo-fermentation and dark fermentation^1^. Based on the technical feasibility considered in terms of stability, simplicity and productivity, the dark fermentation is the technical challenge^2^. Via this successive process, the principal compositions of produced biohydrogen are H_2_ and CO_2_^3^, in which approximately 56% of H_2_ was obtained at 35 °C and 1 atm^4^. A high H_2_ purity of the produced biogas can be obtained using various purification processes such as cryogenic process^5^, absorption^6–8^, membrane separation^6,9–11^ or their combined process^12,13^. Nevertheless, each process exhibits both technical benefits and drawbacks. For example, the cryogenic process exhibits the capability to purify the CO_2_-rich gas stream (CO_2_ ~ 90%) and able to produce liquid CO_2_, but it is the energy intensive and complex^3^. The absorption is able to recover high CO_2_ quantity from a low pressure gas stream with low energy requirement for H_2_ compression; however it requires extensive energy for solvent recovery and has a solvent loss^3,7^. The membrane separation requires the minimal utility in operation and allows an in-situ and continuous flow of biohydrogen, but it gives a low product purity and recovery and also requires the pre-filter/coalescer and/or the gas compression system^3,9^. Another possible process might be the adsorption^14–16^, because of its high efficiency to produce high purity biohydrogen, environmentally friendly, ease of operation and control^3,17–19^.

The biohydrogen purification via the adsorption is carried out based on the concept of the CO_2_ removal from the biohydrogen stream using an effective adsorbent that has fast adsorption kinetics, high adsorption capacity and selectivity as well as high recyclability^20^. Among the promising adsorbents (e.g. zeolites, chalcogenides, metal-organic frameworks (MOF) or resin^21^), the carbonaceous materials exhibit an outstanding property related to a high CO_2_ selectivity compared with H_2_ selectivity^22^. Recently, it was reported that the CO_2_ selectivity of bare ACs was limited by both pore size and average particle size in the presence of high CO_2_ concentration (~ 46.4 mol%), while it was limited by solely average particle size in the presence of low CO_2_ concentration (~14.9 mol%)^23^. A significant CO_2_ reduction in biohydrogen from 46.4% to less than 4.3% was obtained at 10 °C and about 2 MPa using a two-stage adsorption. The resin-derived AC displayed the CO_2_ adsorption capacity of 92.8% from a H_2_/CO_2_ mixture in the presence of 70 mol% CO_2_ at 25 °C and 1.2 bar^24^. The ionic liquid-impregnated activated carbon (IL/AC) was able to adsorb CO_2_ of around 90% from an equimolar H_2_/CO_2_ gas mixture at 30 °C for 6 h^16^. The carbon nanoflake hybrid synthesized from the starch powder in the presence of metal oxide and amine exhibited the CO_2_ adsorption capacity up to 6.77 mmol/g at 25 °C and 1 atm^25^, attributed to its high surface area and mesopore-micropore morphology. The 12 wt.% chitosan-impregnated palm shell-based activated carbon (CHI/AC) completely adsorbed CO_2_ from a CO_2_/H_2_/N_2_ mixture (50:30:20 by volume) up to at least 5 cycles under the adsorption pressure of 4 bar at 298 K^14^, due to the presence of appropriate nitrogen containing surface functional groups on the surface of AC. The impregnation of KOH, KI, CuSO_4_, Na_2_CO_3_, and ZnC_4_H_6_O_4_ on the AC surface importantly improved the CO_2_ adsorption capacity of AC^26^. Among all explored chemical-impregnated AC, the zinc acetate impregnated AC exhibited the highest CO_2_ adsorption capacity of 63.61 mg/g AC at 1 bar, because of the existence of abundant nano-porosity and high BET surface area^26^.

As described above, it can presume that the effectiveness of the biohydrogen purification depends significantly on types and properties of adsorbents. For economic and sustainability perspectives, the synthesis of AC from biomass or wastes (e.g. agriculture-, household- or industry wastes) is the key issue because of their plentiful and worthwhile. In this work, the spent chopsticks were used as raw materials to synthesize AC due to their massive dumping every day in our country. Based on our published works, it was found that the spent chopstick-derived AC obtained from the chemical activation exhibited higher CO_2_ adsorption capacity than those obtained from the physical activation of around 35.8%^21,27^. This is because the chemical activation can effectively promote the formation of appropriate surface area, porosity as well as the surface functional groups, which were responsible for the CO_2_ adsorption. Based on the acid property of CO_2_, the presence of appropriate quantity of basic sites can promote the CO_2_ adsorption through the acid-base interaction^28,29^. Therefore, the strengthened CO_2_ adsorption capacity of the chopstick-derived AC synthesized by physical activation was conducted by the impregnation of metal oxides on the surface of AC. Effects of various metal oxide types including CaO, SrO, MgO and contents of the best metal oxides on the morphology and CO_2_ adsorption capacity were systematically investigated and reported.

## Methods

### Chemicals and precursor materials

All chemicals employed in this work were analytical or GR grade including gaseous nitrogen (N_2_; 99.999%, Alternative Chem), hydrogen (H_2_; 99.99%, Alternative Chem), carbon dioxide (99.99% CO_2_, Alternative Chem), calcium nitrate tetrahydrate (Ca(NO_3_)_2_⋅4H_2_O, KemAus), strontium nitrate (Sr(NO_3_)_2_, Himedia) and magnesium nitrate hexahydrate (Mg(NO_3_)_2_⋅6H_2_O, PanReac Applichem). The carbonaceous material employed in this work was the spent disposable wooden chopsticks. Prior to utilization, they were crushed by a knife mill and sieved to get an average particle size between 0.21 and 4.76 mm. Their dry basis compositions were displayed in our previous published work^27^.

### Preparation of AC and metal oxide-impregnated AC

**Figure 1:**
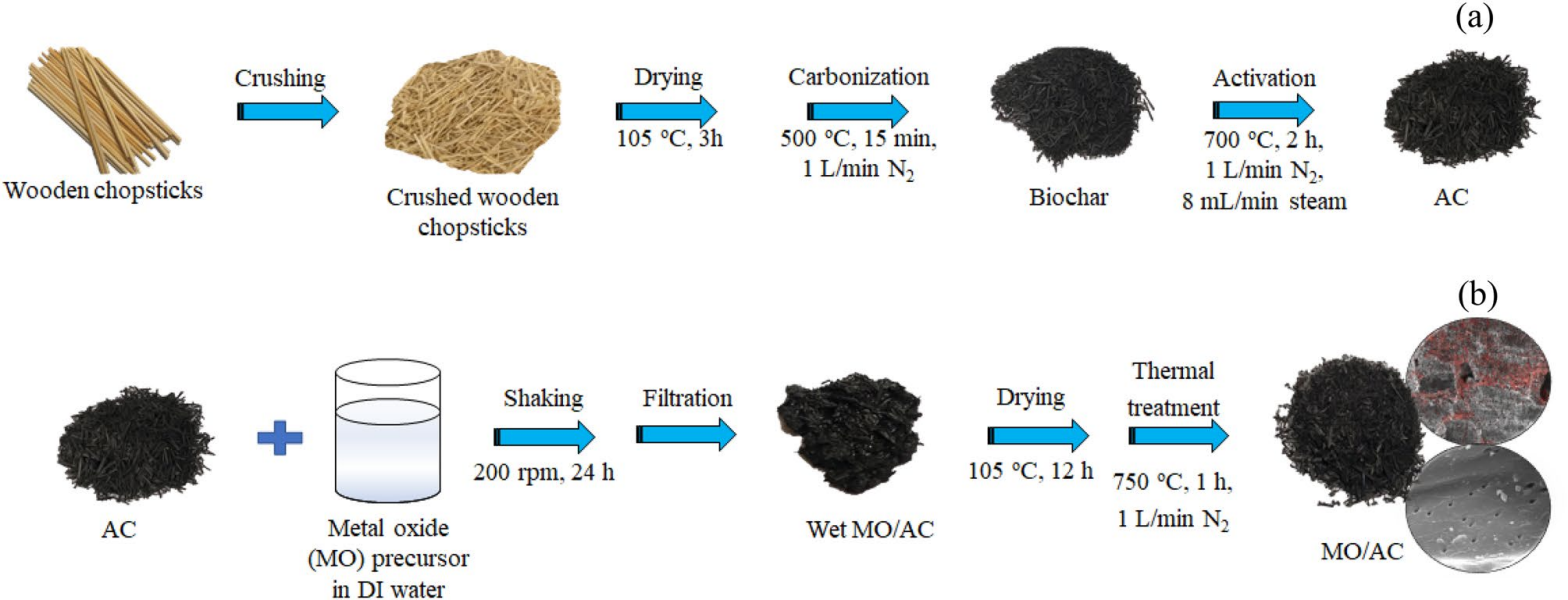
Synthesis procedure of (**a**) AC and (**b**) MO/AC in this work.

A sequential process of carbonization and steam activation was carried out to synthesize ACs from spent disposable wooden chopsticks for the biohydrogen purification application (Fig. 1a). As brief, the crushed spent disposable wooden chopsticks were first dried in air at 105 °C for 3 h to eliminate some free moisture and then subjected to carbonize in a cylindrical stainless-steel reactor under the N_2_ atmosphere at a constant flow rate of 1 L/min. The carbonization temperature and time were respectively fixed at 500 °C and 15 min with a heating rate of 10 °C/min^27^. When the carbonization was complete, the obtained biochar was then carried out to the activation process using steam as the physical activator. The activation process was carried out at 700 °C for 2 h in a horizontal fixed bed reactor under N_2_ atmosphere (1 L/min) using a steam flow rate of 8 mL/min^27^, yielding the chopstick-derived AC denoted as AC.

To strengthen the CO_2_ adsorption capacity of the AC, the metal oxide-impregnated ACs (*x*MO/ACs; *x* is the weight percent of respective impregnated metal oxides), the procedure proposed by Hidayu^30^ was adopted as shown in Fig. 1b. Initially, a certain quantity of Ca(NO_3_)_2_⋅4H_2_O was dissolved in 50 mL deionized (DI) water and added by 4 g of ACs. The slurry was rigorously shaken via the orbital shaker (CTL, Model SK3) at 200 rpm at room temperature for 24 h. Then, the solid portion was separated from the aqueous solution by the vacuum filtration and dried overnight in oven (Binder, Model ED115) at 105 °C. The obtained dried solid was then thermal treated in a circular stainless-steel reactor under N_2_ atmosphere (1 L/min) at 750 °C for 1 h with a constant heating rate of 10 °C/min, getting *x* wt.% of CaO impregnated ACs (*x*CaO/AC). The similar preparation procedure was carried out for SrO and MgO using Sr(NO_3_)_2_ and Mg(NO_3_)_2_⋅6H_2_O as the chemical precursors, respectively.

### Characterization

The physicochemical characteristics of all synthesized spent disposable wooden chopsticks-derived AC were determined as follows. High-resolution image and elemental identification were determined by scanning electron microscopy and energy dispersive X-ray spectrometry (SEM-EDX; IT-500HR JEOL). The presence of chemical bonding and functional groups was qualitative analyzed by Fourier-transform infrared spectroscopy (FTIR; FT/IT-6800 JASCO). The crystallite structures were identified by X-ray diffractometry (XRD; D2 Phaser, Bruker) and Raman spectroscopy (Perkin Elmer Spectrum GX). The textural properties were explored via N_2_ adsorption isotherms using Multipoint Surface Area Analyzer (Micromeritics, Tristar II3020) using the Brunauer-Emmett-Teller (BET) methods. All samples were first degassed at temperature of 300 °C for 10 h in N_2_ atmosphere and analyzed at temperature of 77 K. The quantity of basic site on the surface of adsorbents was determined via the CO_2_ temperature-programmed desorption (CO_2_-TPD, BELCAT-B). Prior to analyzation, approximately 50 mg was pretreated at 200 °C for 30 min under constant helium (He) flow rate of 30 mL/min. The textural properties of the parental AC were partially taken from our published work^27^.

### Adsorption capacity test

The biohydrogen employed in this work was simulated by mixing the commercial H_2_ and CO_2_ at particular molar ratios in the range of 10–50 mol%. The adsorption capacity of all *x*MO/AC samples was tested via the CO_2_ adsorption from the simulated biohydrogen in a horizontal glass tube reactor having an inside diameter of 8 mm ID and 600 mm length at constant temperature of 25 °C and 1 atm. In each experiment, approximately 2 g of adsorbents was dried at 105 °C for 5 h to eliminate free moisture and then carefully packed in a glass column. The simulated biohydrogen gas with the specific CO_2_ concentration in the range of 10 to 50 mol % was then supplied throughout the system at a constant flow rate of 100 mL/min. The flow rate of biohydrogen was precisely controlled by mass flow controllers (S48-2-HMT, Horiba). As the adsorption was proceeded, the concentration of outlet gas stream was analyzed by gas chromatography (GC, Shimadzu GC-8A) with a thermal conductivity detector (TCD) at the INJ/DET temperature of 120 °C, column temperature of 100 °C, and current of 100 mA.

The quantity of adsorbed CO_2_ via the as-synthesized adsorbents expressed in terms of mg CO_2_ per gram of bulk adsorbent was calculated according to Eq. (1)^27^. All reported adsorption capacities were the averaged values obtained from at least three trials with the relative errors of less than 3%.

1 $$q\, = \,\frac{1}{{wM_{W} }}\int\limits_{0}^{t} {\left( {C_{in} - C_{out} } \right)dt} .$$

### Reusability test

The reusability of used adsorbents was carried out by a low temperature thermal treatment. After the fresh adsorbent was used to adsorb 50 mol% CO_2_ from the simulated biohydrogen at a total flow rate of 100 mL/min at 25 °C and 1 atm, it was carefully taken from the adsorption reactor and subjected to the thermal treatment in air at temperature of 105 °C and ambient pressure. Afterward, the regenerated adsorbent was introduced to adsorb CO_2_ from the simulated biohydrogen at identical adsorption condition. The repetitive adsorption/desorption cycles were carried out for 6 times and the quantity of adsorbed CO_2_ on each regenerated adsorbent was determined according to Eq. (1).

## Results and discussion

### Effect of impregnated MO type

**Figure 2:**
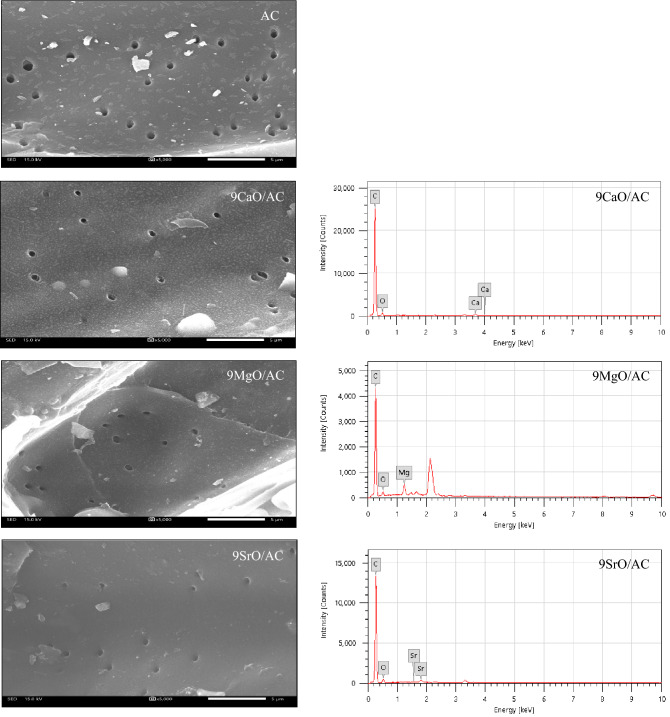
Representative SEM images at magnification of 5000x (left) and EDX (right) of the AC and 9MO/AC samples.

The surface morphology of parental AC and 9MO/AC samples with the magnification of 5000x were displayed in Fig. 2 (left). The AC samples showed a clear and smooth surface with almost uniform dispersion of circular pores. All the 9MO/AC samples exhibited noticeably low appearance pores compared with those of the original AC sample. Besides, they displayed the presence of impregnated MO as clearly confirmed by the EDX images as shown in Fig. 2 (right), thereby causing the blurriness surface.

**Figure 3:**
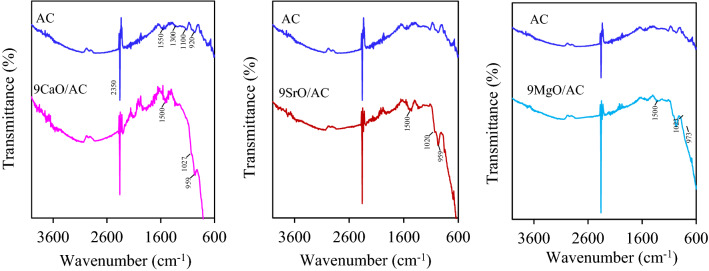
Representative FTIR spectra of the AC^27^ and 9MO/AC samples.

Figure 3 shows the FTIR spectra of the parental AC and 9MO/AC samples. The FTIR spectrum of the parental AC showed peaks of aromatic C–H out-of-plane bending mode at a wavenumber lower than 920 cm^−1^^31^, C–O stretching vibration of surface groups such as ethers, esters, cyclic ethers, lactonic groups, phenolic groups, and also carboxylic acids and cyclic anhydrides at wavenumber of 920–1300 cm^−1^^32^ and C=C stretching vibration of sp2 hybridized carbon at wavenumber of 1480–1650 cm^−1^. Intense spectra appeared a wavenumber of 2350 cm^−1^ due to atmospheric CO_2_^33,34^. The FTIR spectra of the 9MO/ACs exhibited almost similar band position with respect to the parental AC with a more intense band of C–O stretching vibration of ether at 920–1100 cm^-1^ and polyaromatic C=C stretching vibration of sp^2^ hybridized carbons at 1500 cm^−1^^32^. Unlike the EDX analysis, the presence of impregnated MO was not obviously observed.

**Figure 4:**
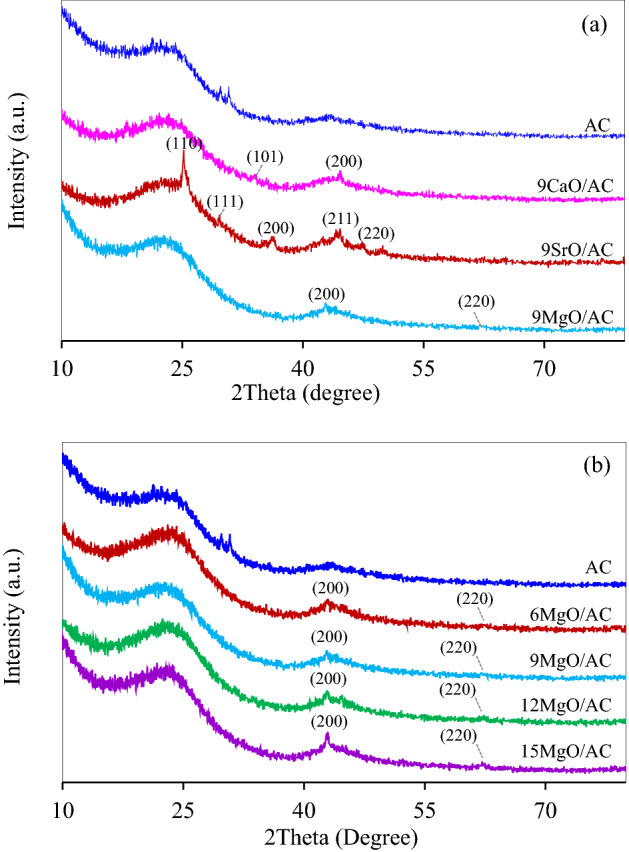
Representative XRD pattern of (**a**) 9MO/AC and (**b**) *x*MgO/AC samples.

Figure 4a presented the XRD spectra of the 9MO/AC samples together with the AC sample. It was obviously seen that the original AC displayed two broad diffraction peaks at 2θ of 20–30° and 40–50°, due to the presence of amorphous carbon structure generated from the disordered carbon rings^35,36^. Two spike peaks observed at 2θ of 29.76° and 30.79° could be due to the presence of oxide species of raw material-contained minerals such as K_2_O species (JCPDS card no. 26-1327)^37^. However, they were disappeared after the metal oxide impregnation, probably due to the reduction of K_2_O with carbon (C) at the temperature higher than 700 °C^38^. The XRD spectra of 9CaO/AC exhibited a well-defined peak at 2θ of 44.63°, assigning to the crystal plane of CaO as (200) (JCPDS card no. 77-2376)^39^. Besides, additional peak appeared at 2θ of 34.02° corresponding to the presence of Ca(OH)_2_ as (101) plane (JCPDF 1-1079) was observed, indicating a low stability of crystal CaO structure^40^. The diffraction peaks of 9SrO/AC sample exhibited the characteristic peaks of SrO crystal planes as (110), (111), (200) and (220) at 2θ of 25.6°, 26.8°, 35.6° and 50.2°, respectively (JCPDS card no. 01–075-0263)^41,42^. The presence of Sr(OH)_2_ was also found in the 9SrO/AC structure, diagnosing from the peak at 2θ of 44.6° which was corresponding to the (211) plane of Sr(OH)_2_^42^. The XRD patterns of 9MgO/AC samples illustrated two diffraction peaks at 42.9° and 62.3°, indicating the (200) and (220) lattice planes of the MgO cubic structure (JCPDS card no. 4-829)^43,44^. The average crystallite sizes of all impregnated MO were then estimated from the full width at half maximum (FWHM) of the peaks (200), (110) and (200) planes for CaO, SrO and MgO, respectively, using the Scherrer equation (Eq. (2))^45^. As listed in Table 1, the 9CaO/AC sample exhibited the largest average CaO crystallite size of 47.2 nm, while the 9MgO/AC sample showed the smallest average MgO crystallite size of 9.3 nm, which might play the role on their textural property.

2 $$D^{{}} =^{{}} \frac{K\lambda }{{\beta \cos \theta_{B} }}$$

**Table 1:** Effect of metal oxide types and loading on textural properties and basic site density.

Sample	Crystallite size of MO (nm)	*I*_*G*_/*I*_*D*_	Textural property				CO_2_ desorption peak (°C)	Basic site density (mmol/g)	Equilibrium CO_2_ adsorption (mg/g)**
			*S*_BET_ (m^2^/g)	*S*_t-plot_ (m^2^/g)	*V*_mic_ (cm^3^/g)	*V*_mes_ (cm^3^/g)			
AC^27^		1.45	330.0	263.9	0.1484	0.0375	114.6	0.007	85.19 ± 1.79
9CaO/AC	47.2	1.09	217.2	189.0	0.1067	0.0183	110.3	0.019	84.20 ± 2.86
9SrO/AC	27.1	1.02	227.5	198.9	0.1073	0.0489	114.2	0.021	87.37 ± 2.67
9MgO/AC	9.3	1.06	234.4	207.4	0.1171	0.0171	122.3	0.025	89.46 ± 1.38
6MgO/AC	2.7	1.01	223.4	187.15	0.1063	0.0221	n.a.*	n.a.	87.49 ± 0.21
9MgO/AC	9.3	1.06	234.4	207.4	0.1171	0.0171	122.3	0.025	89.46 ± 1.38
12MgO/AC	13.8	1.05	284.0	238.8	0.1355	0.0269	105.7	0.025	94.02 ± 0.65
15MgO/AC	17.8	1.10	247.8	215.5	0.1219	0.0194	121.7	0.026	87.13 ± 7.02

^*^Not analyzed.

^**^Equilibrium adsorption in the presence of 50 mol% CO_2_ in the biohydrogen at a total flow rate of 100 mL/min, 25 °C and 1 atm.

**Figure 5:**
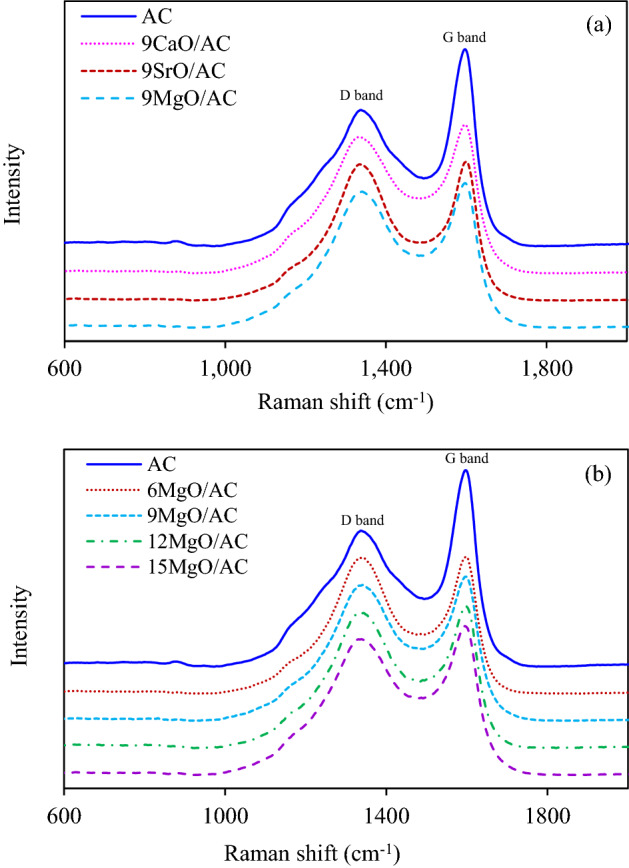
Raman spectra of (**a**) 9MO/AC and (**b**) *x*MgO/AC samples.

To intensively explore the crystallographic structure of all *x*MO/AC samples, the Raman spectra was then carried out. As shown in Fig. 5a, two spectra were observed for all synthesized 9MO/AC samples at 1336 and 1597 cm^−1^, corresponding to the D band and G band of the graphitic carbon, respectively^33^. Typically, the D band depicts the A1g breathing mode, which represents the disordered and defective structures, while the G band corresponds to the E2g symmetry modes, which represents the presence of high crystallite and ordered structure of graphitic carbon^20,46^. For all samples, the D band intensity were close to the G band intensity, indicating the presence of high defective or disordered structures in all synthesized ACs. As recently reported, the adsorbents with high defective structures normally exhibited a high surface area and porous structure, which could strongly interact with the gas adsorption capacity^47^. Among all adsorbents, the AC exhibited a sharper and higher spectrum of both D band and G band than other samples, suggesting its high crystallite structures compared with other adsorbents. Quantitatively, the intensity ratio of G band and D band (*I*_*G*_/*I*_*D*_) of all samples was estimated and tabulated in Table 1. It can be obviously seen that the AC exhibited the highest value of *I*_*G*_/*I*_*D*_ of 1.45, indicating its high crystallite and ordered structure compared with other samples. Interestingly, all 9MO/AC samples exhibited comparable values of *I*_*G*_/*I*_*D*_ in the range of 1.02–1.09, which were lower than that of unimpregnated AC. This suggested that the impregnation of metal oxides including CaO, SrO and MgO increased the defective and disordered structures of the resultant ACs. However, different types of impregnated metal oxides did not significantly change the crystallographic structure of all *x*MO/AC samples.

**Figure 6:**
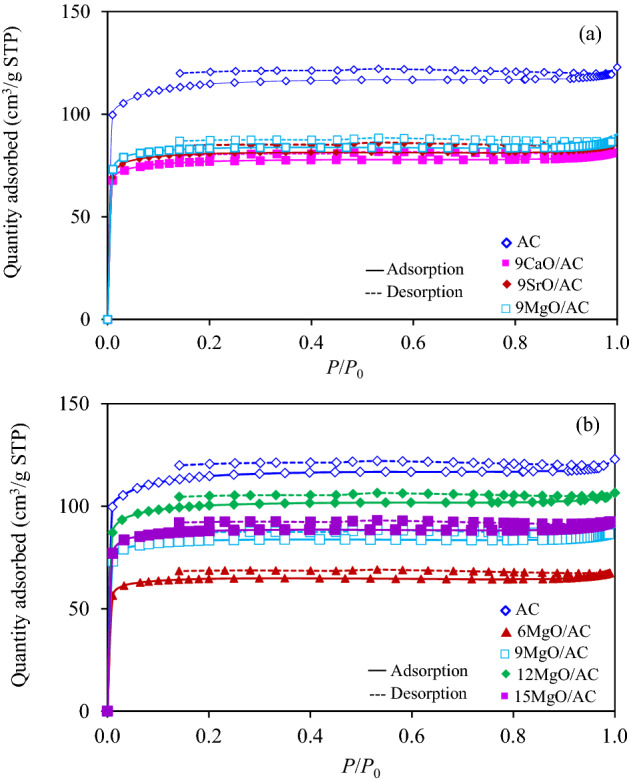
N_2_ adsorption/desorption isotherms of (**a**) 9MO/AC and (**b**) *x*MgO/AC samples.

Regardless of the textural property, all 9MO/AC samples exhibited the Type I(b) isotherm of N_2_ adsorption/desorption (Fig. 6a) according to the recent IUPAC classification^48^. Impregnation of CaO, SrO and MgO diminished the surface area and differential pore volumes of the parental AC as shown in Table 1. The decreased surface area and pore volume in the presence of respective MO was probably due to the deposition of MO on the walls and/or pore blocking^49^. Among all samples, the 9MgO/AC showed the best textural property considered in terms of high surface area and pore volume. This might be due to the presence of small crystallite size of MgO particles that required lower impregnated surface of AC as well as a pore blockage, thus providing higher available area for N_2_ adsorption. Another possible reason is due to the catalytic effects of Mg ions in MgO, which can energize at high temperature and intercalate into the carbon matrix to create new pores in carbon-based materials^50^.

**Figure 7:**
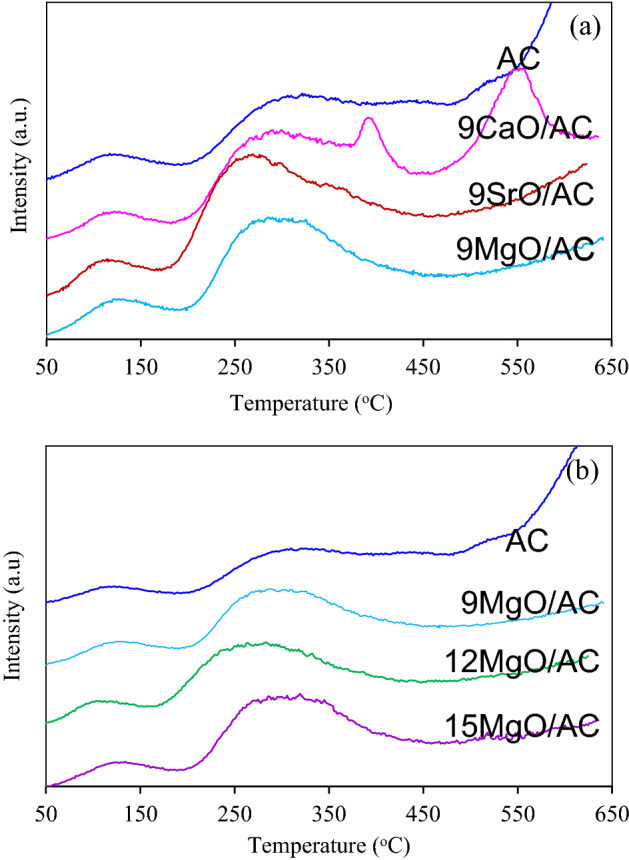
CO_2_-TPD of (**a**) 9MO/AC and (**b**) *x*MgO/AC samples.

The number of basic sites of as-synthesized AC samples was then estimated using the CO_2_-TPD under the same analysis condition. As shown in Fig. 7a, with the exception of 9CaO/AC, two desorbed peaks were detected at temperatures below 200 °C and temperature between 200 and 500 °C, which were attributed to the desorbed CO_2_ and/or decomposition of weak- and medium basic sites, respectively^51^. The amount of desorbed CO_2_ can be computed from the integrated peaks area at the temperature below 200 °C after the blank- and based line corrections according to Eq. (3)^52^.

3 $$q_{{{\text{CO}}_{{2}} }} \, = \,\frac{{\sum {{\text{TCD}}{}_{{{\text{signal}}}}\, \times \,C_{{\text{A}}} } }}{w}$$

As summarized in Table 1, the AC exhibited the basic site density of 0.007 mmol/g, which was 2.7–3.6 times lower than that of 9MO/AC samples. Different types of impregnated MO displayed a diversity in basic site density, which can be ranked as the order of 9MgO/AC > 9SrO/AC > 9CaO/AC.

The equilibrium CO_2_ adsorption of all synthesized 9MO/AC adsorbents in the presence of 50 mol% CO_2_ in the biohydrogen at 25 °C and 1 atm was summarized in Table 1. It is noteworthy to note that all impregnated AC exhibited comparable or higher CO_2_ adsorption capacity than that of un-impregnated one, although they had a worse surface property considered in terms of surface area and pore volume. This suggests that the basic site also played a crucial role on the CO_2_ adsorption via the acid-basic interaction according to the acid property of CO_2_^53^. That is, the unpaired electrons of the basic surface functional groups can initiate a well interaction with the CO_2_ molecules, resulting to the enhancement of CO_2_ adsorption via the chemical adsorption^21^. Among all synthesized 9MO/AC samples, the 9MgO/AC exhibited the highest CO_2_ adsorption capacity of 89.46 mg/g at identical adsorption condition. This is probably due to the synergetic effect of textural property and surface chemistry of the 9MgO/AC that can promote the CO_2_ adsorption capacity.

### Effect of impregnated MgO content

**Figure 8:**
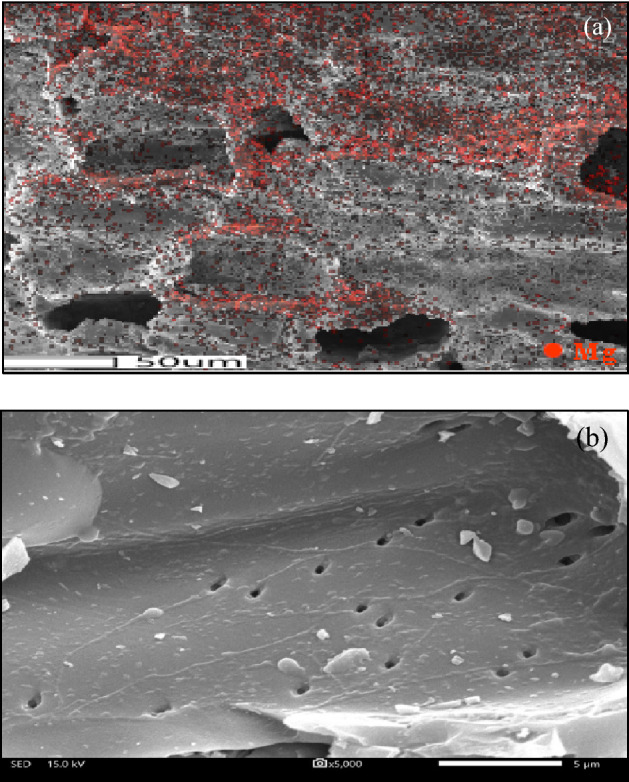
Representative (**a**) metal distribution and (**b**) SEM image of 12MgO/AC.

The effect of impregnated MgO contents on the surface morphology of AC was first explored in this part. The *x*MgO/AC samples with different MgO contents in the range of 6–15 wt.% exhibited almost similar external features with respect to the parental sample (figure not shown). As shown in Fig. 8, an acceptable distribution of MgO nanoparticles on the parental AC was observed together with some well-developed pores.

The XRD spectra of as-synthesized *x*MgO/AC samples were also depicted in Fig. 4b. Apart from the broad peaks of amorphous carbon at 2θ of 20–30^o^ and 40–50°, all *x*MgO/AC samples exhibited the main characteristic peaks of MgO cubic structure at 2θ of 42.9° and 62.3°, corresponding to the crystal planes of (200) and (220). The XRD intensity of these peaks increased as the increase of MgO content. Via the Scherrer equation, the average crystallite sizes of all impregnated MgO were quantitative estimated and summarized in Table 1. As expected, their average crystallite sizes increased as the increased MgO contents, probably due to the agglomeration of MgO nanoparticles on the AC surface.

The effect of MgO content on the crystallographic structure of AC was also explored using Raman spectra. As shown in Fig. 5b, a typical Raman spectrum was observed for all *x*MgO/AC samples. That is, the D band spectrum was observed at 1336 cm^-1^, while the G band spectrum was observed at 1597 cm^-1^. As summarized in Table 1, all *x*MgO/AC samples exhibited a considerably low values of *I*_*G*_/*I*_*D*_ compared with the parental AC, indicating their low crystallinity or, in other words, their high disordered structure of graphitic carbon. Besides, it can be noticed that the impregnation of MgO in the range of 6–15 wt.% affected slightly the crystallographic structure of the obtained samples.

Regarding the effect of impregnated MgO on the textural property, the addition of MgO at all contents decreased the intensity of N_2_ adsorption/desorption isotherms compared with the parental AC as depicted in Fig. 6b. The quantitative values of surface area and pore volume of all *x*MgO/AC samples were summarized in Table 1. Among all *x*MgO/AC samples, the 12MgO/AC samples exhibited the highest surface area and pore volume. This suggested that an appropriate content of impregnated MgO (~12 wt.%) exhibited an effective energetic ability to create some pores as well as surface area in the carbon matrix. Although too high MgO content (e.g. 15 wt.% in this case) might exhibit a higher energetic ability to create some pores, it could possibly induce the pore blockage by the MgO itself, which in turn reduced the textural property. The CO_2_-TPD of *x*MgO/AC samples also displayed the desorbed CO_2_ and/or decomposition peaks of weak and medium basic sites as shown in Fig. 7b. On the weight basis, the comparable basic site density was obtained for all *x*MgO/AC samples as listed in Table 1, suggesting that the impregnation of MgO in the range of 9–15 wt.% slightly affected the basic site density of the obtained adsorbents. The CO_2_ adsorption capacities of all *x*MgO/AC samples from the biohydrogen with 50 mol% CO_2_ at a total flow rate of 100 mL/min, temperature of 25 °C and pressure of 1 atm were measured and displayed in Table 1. As expected, the 12MgO/AC sample exhibited the highest CO_2_ adsorption capacity of 94.02 mg/g, which was higher than the unimpregnated AC of around 10.4%. This is attributed to its high textural properties and basic site density.

**Figure 9:**
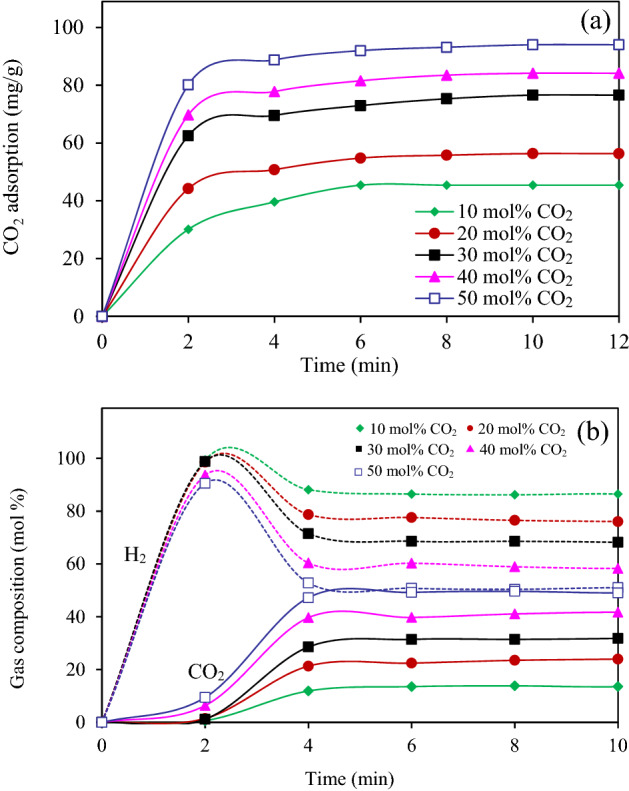
(**a**) CO_2_ adsorption capacity of 12MgO/AC from the biohydrogen and (**b**) exit gas concentrations at different CO_2_ concentrations in the range of 10–50 mol% at a total flow rate of 100 mL/min, 25 °C and 1 atm.

Figure 9a displays the effect of CO_2_ concentrations in the biohydrogen on the CO_2_ adsorption capacity of the 12MgO/AC. It was found that the CO_2_ adsorption increased as the progress of adsorption time and reached a plateau after 4–6 min. Besides, the CO_2_ adsorption capacity increased as the increase of CO_2_ concentrations in the mixture. This is attributed to the fact that a high concentration gradient between bulk phase and adsorbent surface can initiate a high driving force for CO_2_ transfer to the adsorption site, and consequently improved the adsorption capacity of adsorbent^54^. Quantitatively, the equilibrium adsorptions of the 12MgO/AC were around 45.41, 56.32, 76.58, 84.16 and 94.02 mg/g in the presence of CO_2_ in the biohydrogen of 10, 20, 30, 40 and 50 mol%, respectively. Concentrations of CO_2_ and H_2_ in the outlet gas stream leaving the adsorption process was depicted in Fig. 9b. A well separation between CO_2_ and H_2_ was observed at the early adsorption period. Almost pure H_2_ was evolved during the first 2–4 min and the CO_2_ was emitted afterward. The breakthrough time of CO_2_ exhibited a direct proportion to the CO_2_ concentration in the inlet stream. That is, the gas stream with a high CO_2_ concentration displayed a faster breakthrough time than that with a low CO_2_ concentration. For a gas mixture with 50 mol% CO_2,_ the as-synthesized 12MgO/AC was able to capture CO_2_ from the biohydrogen and allowed the production of the H_2_-rich stream with the H_2_ purity up to 90% for the first 2 min of adsorption period. This phenomenon allows the use of the proposed material as an adsorbent for biogas purification in the solid-gas reactor such as the circulating fluidized bed reactor because this reactor effectively work even through it has a short contact time^21^.

Table 2 summarizes the biohydrogen purification by adsorption via several types of adsorbents at 25 °C. It can be noticed that the ability of the biohydrogen purification by the concept of CO_2_ removal varied from task to task. For instance, the hybridized N-doped porous AC and Fe_2_O_3_ (NDPAC/Fe_2_O_3_) exhibited the highest CO_2_ adsorption capacity of 297.9 mg/g, while the zinc acetate -impregnated AC (ZnAC_2_/AC) showed the adsorption capacity of 63.61 mg/g. The variation in CO_2_ adsorption capacity might be attributed to the differences of adsorbent property coming from the utilization of different raw materials, synthesis methods or impregnated substances as well as conditions and reactors used to purify the biohydrogen. Nevertheless, the CO_2_ adsorption capacity of the 12MgO/AC synthesized in this work was on par to those reported previously.

**Table 2:** Comparative biohydrogen purification by adsorption methods.

Adsorbent	Reactor	Purification condition				CO_2_ adsorption (mg/g)	Reference
		Temperature (°C)	Pressure (atm)	Gas flowrate (mL/min)	Feed gas (H_2_ : CO_2_ : N_2_)		
Resin-based AC	Fixed bed	25	1.18	50	30 : 70 : 0	92.83	^24^
Olive stone-based AC	Adsorption column	25	1.0	40	30 : 70 : 0	110.0	^59^
CHI/AC	Fixed bed	25	3.95	2000	30 : 50 : 20	165.5	^14^
NDPAC/Fe_2_O_3_	Fixed bed	25	1.0	50	40 : 50 : 0	297.9	^25^
ZnAC_2_/AC	Fixed bed		0.99	250	50 : 50 : 0	63.61	^26^
12MgO/AC	Fixed bed	25	1.0	100	50 : 50 : 0	94.0	This work

**Figure 10:**
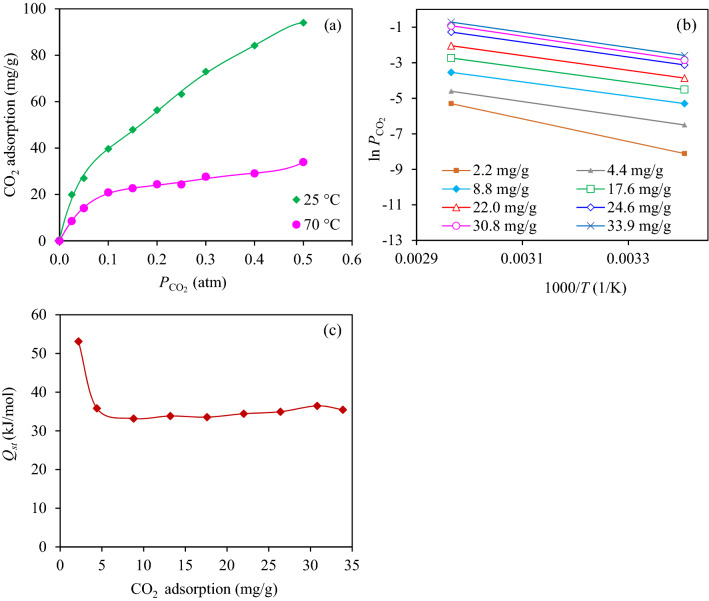
(**a**) CO_2_ adsorption isotherm, (**b**) isosteric adsorption and (**c**) variation of isosteric heat of adsorption of 12MgO/AC at 1 atmosphere.

Due to the CO_2_ adsorption by AC is the heterogeneous in nature^55^, the interactive energy between adsorbate and adsorbent is not constant^56^. Therefore, the estimation of the perception of the isosteric heat of adsorption might benefit for the thermal management of the adsorption process. Figure 10a displays the adsorption isotherm of CO_2_ of the synthesized 12MgO/AC at 25 and 70 °C and 1 atm. As expected, a high CO_2_ adsorption was well achieved at low temperature due to the exothermic behavior of the CO_2_ adsorption process. From these isotherms, the value of isosteric heat of adsorption can be estimated from the Clausius-Clapeyron equation (Eq. (4))^56^.

4 $$Q_{st} \, = \,RT^{2} \left[ {\left( {\frac{{\partial \left( {\ln P} \right)}}{\partial T}} \right)} \right]_{c}$$

As shown in Fig. 10b, plots of $\ln P_{{{\text{CO}}_{2} }}$ versus the reciprocal absolute temperature showed a linear line with negative slope, which allowed to calculate the isosteric heat of adsorption. The variation of isosteric heat of adsorption against the adsorbed CO_2_ quantity was displayed in Fig. 10c. It can be noticed that high values of isosteric heat were observed at low surface coverages, indicating a strong adsorbate-adsorbent interaction^57^. A gradual decrease of isosteric heat was observed as the increase of amount adsorbed CO_2_ and reached a plain when the adsorbed CO_2_ was higher than 4.4 mg/g. This might be due to the limitation of available active site to interact with the CO_2_ molecules, leading to a weak interaction between them. Based on the obtained results, the isosteric heat of adsorption at high adsorbed CO_2_ was slightly fluctuate in narrow range with the average value of 34.7 ± 1.16 kJ/mol, which was higher than a pure physical adsorption (< 20 kJ/mol), but lower than a pure chemical adsorption (90–100 kJ/mol)^58^. This suggested that the adsorption mechanism of CO_2_ on the 12MgO/AC was a combined mechanism of physisorption and chemisorption.

**Figure 11:**
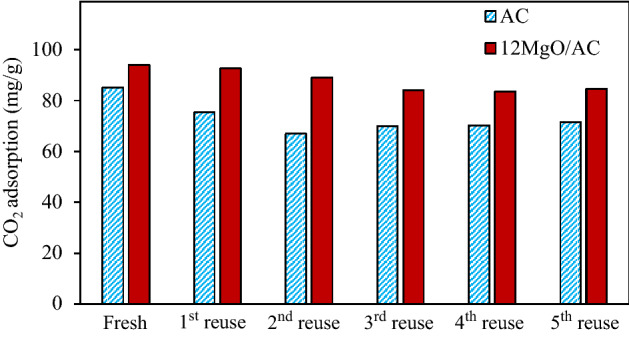
Benchmarking of CO_2_ adsorption capacity 25 °C and 1 atm between 12MgO/AC and unimpregnated AC^27^ after the thermal regeneration.

The reusability of the best adsorbent (12MgO/AC) was also explored and benchmarked with the parental AC or the unimpregnated chopstick-derived AC reported in our previous work^27^. As shown in Fig. 11, the CO_2_ adsorption capacity of both adsorbents decreased as the increased adsorption cycles. This suggests that the adsorption mechanism importantly affected the regeneration efficiency. That is, the proposed regeneration procedure can effectively remove only some weak and/or medium adsorbed CO_2_. However, it cannot effectively dispose some strong adsorbed CO_2_. At the 5th reuse, the drops of CO_2_ adsorption capacity of the parental AC and 12MgO/AC were around 15.96 and 9.96%, respectively.

## Conclusion

The strengthened CO_2_ adsorption of the spent chopstick-derived ACs was carried out via the impregnation of several metal oxides including CaO, SrO and MgO on their surface. The preliminary results depicted that the impregnation of such metal oxides affected the crystallographic structure, textural property, basic site density as well as the CO_2_ adsorption capacity of the obtained adsorbents. The crystallographic structure insignificantly affected the CO_2_ adsorption capacity, while both textural property and basic site density played a crucial role. Among all MO/AC samples, the 12MgO/AC exhibited the highest CO_2_ adsorption capacity compared with other synthesized adsorbents. A high purity biohydrogen of greater than 90 mol% H_2_ can be achieved via this successive adsorbent in a high CO_2_ concentration (~50 mol%). The adsorption mechanism occurred via a combined process of the physisorption and chemisorption. The lessening of CO_2_ adsorption capacity of around 9.96% was observed via the best adsorbent over the six adsorption/desorption cycles.

## Data Availability

All data generated or analyzed during this study are included in this published article.

## List of symbols

*C*_*A*_: Calibration factor (mmol CO_2_/area unit)
*C*_*in*_: Inlet concentration of CO_2_ (mmol/min)
*C*_*out*_: Outlet concentration of CO_2_ (mmol/min)
*D*: Average crystallite size (nm)
*K*: Dimensionless shape factor (0.94)
*M*_*W*_: Molecular weight of CO_2_ (g/mol)
*P*: Pressure of CO_2_ (atm)
*q*: CO_2_ adsorption capacity of adsorbent (mg/g)
$q_{{{\text{CO}}_{2} }}$: Amount of CO_2_ (mmol/g)
*Q*_*st*_: Isosteric heat of adsorption (kJ/mol)
*R*: Gas constant (8.314 J/molK)
*t*: Adsorption time (min)
*T*: Absolute temperature (K)
*w*: Amount of adsorbent (g)

## Greek letters

*β*: Half-height peak width in radians
*λ*: X-ray wavelength (0.15406 nm)
*θ*_*B*_: Bragg angle corresponding to the peak maximum
